# Brain Overexpression of Uncoupling Protein-2 (UCP2) Delays Renal Damage and Stroke Occurrence in Stroke-Prone Spontaneously Hypertensive Rats

**DOI:** 10.3390/ijms21124289

**Published:** 2020-06-16

**Authors:** Carla L. Busceti, Maria Cotugno, Franca Bianchi, Maurizio Forte, Rosita Stanzione, Simona Marchitti, Giuseppe Battaglia, Ferdinando Nicoletti, Francesco Fornai, Speranza Rubattu

**Affiliations:** 1 IRCCS Neuromed, 86077 Pozzilli, Italy; clbusceti@libero.it (C.L.B.); maria.cotugno@neuromed.it (M.C.); franca.bianchi@neuromed.it (F.B.); maurizio.forte@neuromed.it (M.F.); stanzione@neuromed.it (R.S.); simona.marchitti@neuromed.it (S.M.); giuseppe.battaglia@neuromed.it (G.B.); ferdinandonicoletti@hotmail.com (F.N.); francesco.fornai@med.unipi.it (F.F.); 2 Department of Physiology and Pharmacology “V. Erspamer” University Sapienza of Rome, 00185 Rome, Italy; 3 Department of Translational Research and New Technologies in Medicine and Surgery, University of Pisa, 56126 Pisa, Italy; 4 Department of Clinical and Molecular Medicine, School of Medicine and Psychology, Sapienza University of Rome, 00189 Rome, Italy

**Keywords:** UCP2, mitochondria, brain, stroke, renal damage, stroke-prone spontaneously hypertensive rat

## Abstract

The downregulation of uncoupling protein-2 (UCP2) is associated with increased brain and kidney injury in stroke-prone spontaneously hypertensive rats (SHRSP) fed with a Japanese style hypersodic diet (JD). Systemic overexpression of UCP2 reduces organ damage in JD-fed SHRSP. We examined the effect of brain-specific UCP2 overexpression on blood pressure (BP), stroke occurrence and kidney damage in JD-fed SHRSP. Rats received a single i.c.v. injection of a lentiviral vector encoding UCP2 (LV-UCP2), or an empty vector. The brain delivery of LV-UCP2 significantly delayed the occurrence of stroke and kidney damage. The large reduction of proteinuria observed after LV-UCP2 injection was unexpected, because BP levels were unchanged. At the time of stroke, rats treated with LV-UCP2 still showed a large UCP2 upregulation in the striatum, associated with increases in OPA1 and FIS1 protein levels, and reductions in PGC1-α, SOD2, TNFα mRNA levels and NRF2 protein levels. This suggested UCP2 overexpression enhanced mitochondrial fusion and fission and reduced oxidative damage and inflammation in the striatum of JD-fed SHRSP rats. Our data suggest the existence of central mechanisms that may protect against hypertension-induced organ damage independently of BP, and strengthen the suitability of strategies aimed at enhancing UCP2 expression for the treatment of hypertensive damage.

## 1. Introduction

Uncoupling protein-2 (UCP2) is a mitochondrial anion carrier protein, which uncouples oxidative phosphorylation from ATP production by dissipating the proton gradient generated across the inner mitochondrial membrane [1,2,3]. UCP2 is protective against oxidative damage by reducing the amount of reactive oxygen species (ROS), produced during oxidative phosphorylation by the electron transport chain [4,5,6]. Hence, a reduced UCP2 expression has deleterious effects on cell viability, favors tissue damage, and may contribute to the pathophysiology of cell death in several disorders [1,7,8]. Recent studies underscored the fine molecular mechanisms that link UCP2 to cell resilience in renal and cardiac diseases [9,10,11,12]. UCP2 gene variants were found to be associated with reduced insulin sensitivity and obesity [13], metabolic syndrome [14], prediabetes and type 2 diabetes mellitus [15,16], hypertension [17], coronary artery disease [18], and stroke outcome [19]. This supports data obtained in cellular and experimental animal models and suggests that UCP2 is a potential therapeutic target for major metabolic and cardiovascular diseases [1].

In our previous studies we found a reduced brain UCP2 expression associated with an up-regulation of the UCP2-targeting miR503 in stroke-prone spontaneously hypertensive rats (SHRSP) exposed to a Japanese style hypersodic diet (JD) [10,20,21,22,23]. A causal link between lower UCP2 levels and the pathological phenotype of JD-fed SHRSP rats was suggested, but not proven, by pharmacological studies, with the peroxisome proliferator activator receptor-α ligands, fenofibrate or *Brassica oleracea* extracts. Both substances up-regulated UCP2 and protected JD-fed SHRSP rats against the development of renal and brain damage and susceptibility to stroke [20,21]. However, a direct evidence that UCP2 is protective in this stroke model is still lacking. We now provide this evidence by overexpressing UCP2 in the CNS with the aid of a lentiviral vector. This approach allowed us to demonstrate, for the first time, that selective UCP2 overexpression in the corpus striatum protects JD-fed SHRSP rats against renal damage and delays stroke occurrence. UCP2 overexpression was also associated with biochemical changes that are indicative of antioxidant and anti-inflammatory effects and the improvement of mitochondrial quality control.

## 2. Results

### 2.1. Brain Overexpression of UCP2 Did Not Affect Body Weight (BW) Gain and BP in SHRSP Rats

We measured BW of all rats treated with either lentiviral vector encoding UCP2 (LV-UCP2) or the empty vector every week, starting from the 2nd week following the onset of the JD. BW values did not differ between the two groups at all time points, although a trend to a reduction was observed in the group of rats treated with LV-UCP2 (Table 1).

Systolic BP was assessed weekly starting from the fourth week of JD. Values ranged from 186 to 226 mmHg in all measurements, as expected in JD-fed SHRSP rats, and were approximately 40 and 60 mmHg greater with respect to age-matched SHRSP not exposed to JD and to normotensive Wistar Kyoto rats, respectively (not shown). BP values were identical in the two groups of JD-fed SHRSP rats treated with LV-UCP2 or empty vector (Table 2).

### 2.2. Early Protection against Proteinuria in Rats Overexpressing UCP2

**Figure 1:**
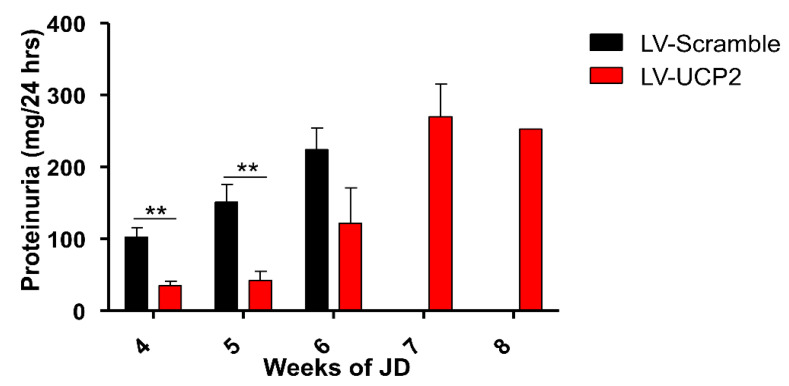
UCP2 brain delivery reduced renal damage in JD-fed SHRSP rats. Proteinuria levels in JD-fed SHRSP rats receiving a single i.c.v. injection of LV-UCP2 (*n* = 6) or the empty vector (LV-Scramble-i.c.v.) (*n* = 6) are shown. Values are means ± S.E.M. ** *p* < 0.001 vs. the corresponding values obtained in rats injected with LV-UCP2 (Student’s *t* test).

Kidney damage is a consistent component of the pathological phenotype of SHRSP rats, and precedes the first behavioral manifestation of stroke of at least three weeks, in response to a hypersodic diet. The assessment of proteinuria showed that brain overexpression of UCP2 caused a substantial protection of early kidney damage in JD-fed SHRSP rats. Proteinuria was largely reduced at four and five weeks in rats treated with LV-UCP2, as compared to their control LV-Scramble-injected rats. At six weeks, there was a smaller difference between the two groups, which did not reach statistical significance (Figure 1). At 7 and 8 weeks, proteinuria was substantial in LV-UCP2-treated rats. Comparisons were not made at these last two time points, because all rats treated with the empty vector had already shown the first episode of stroke, and were therefore excluded from the analysis (Figure 1).

### 2.3. Delayed Onset of Stroke in Rats with Brain Overexpression of UCP2

**Figure 2:**
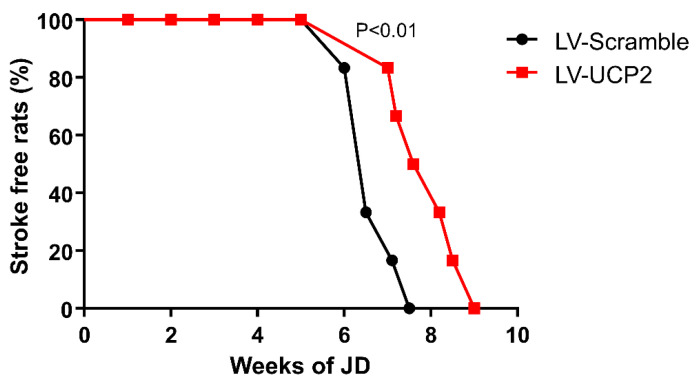
UCP2 brain delivery delayed stroke occurrence in JD-fed SHRSP rats. The graph shows the percentage of stroke-free JD-fed SHRSP rats at different weeks, following a single i.c.v. injection of either LV-UCP2 (*n* = 6) or the empty vector (LV-Scramble) (*n* = 6). For statistical analysis, see paragraph 4.5.

As opposed to classical models of transient or permanent focal ischemia, stroke episodes in JD-fed SHRSP rats are heterogeneous in terms of temporal latency and anatomical distributions of brain microinfarcts. Hence, behavioral analysis is routinely used for the assessment of stroke occurrence in these animals. All JD-fed SHRSP rats in our study showed behavioral manifestations of the first episode of stroke (paresis, paralysis or convulsive seizures), between the 6th and the 9th week of the hypersodic diet. None of the animals had sudden death as a manifestation of the first episode of stroke. For ethical reasons, we did not examine whether brain UCP2 overexpression could prolong the lifespan of JD-fed SHRSP rats, and all animals where euthanized at the time of the first episode of stroke. Treatment with LV-UCP2 delayed the onset of stroke by about 12–14 days with respect to rats treated with the empty vector; the difference between the two groups of rats was highly significant (Figure 2).

### 2.4. Demonstration of Brain UCP2 Overexpression in Rats Treated with LV-UCP2

**Figure 3:**
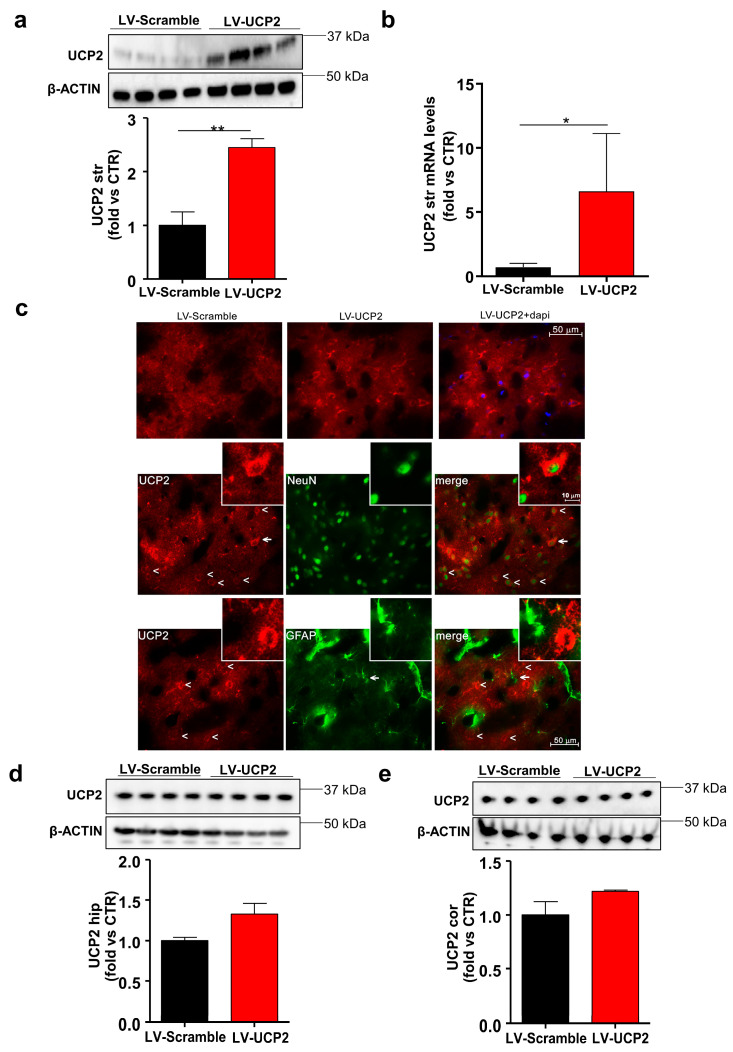
Overexpression of UCP2 in the striatum of JD-fed SHRSP rats, receiving a single i.c.v. injection of LV-UCP2. UCP2 protein and mRNA levels in the striatum of JD-fed SHRSP rats receiving a single i.c.v. injection of LV-UCP2 or the empty vector (LV-Scramble) are shown in (**a**) and (**b**), respectively. Values are means ± S.E.M. of 6 animals per group. * *p* < 0.05; ** *p* < 0.001 (Student’s *t* test). Animals were killed at the time of the first stroke episode. Immunohistochemical analysis of UCP2 in one representative animal from the two groups is shown in (**c**). DAPI was used as blue fluorescent nucleic acid stain. Double fluorescent staining of UCP2 (red) and either NeuN (green) or GFAP (green) is shown in the lower panel. Arrowheads indicate UCP2 immunoreactive cells, whereas arrows indicate cells shown in the insert at higher magnification. UCP2 protein levels in the hippocampus and cerebral cortex of the two groups of animals are shown in (**d**) and (**e**), respectively. Values are means ± S.E.M. (*n* = 6 in both groups).

To demonstrate that a single i.c.v. administration could cause a long-lasting overexpression of UCP2, we first performed an immunoblot analysis in the corpus striatum, hippocampus, and cerebral cortex of rats euthanized at the time of the first episode of the stroke. The temporal disomogeneity with respect to the beginning of the hypersodic diet is inherent to the experimental design, and could have underestimated the difference in UCP2 protein levels between SHRSP rats treated with LV-UCP2 and those treated with the empty vector (the latter were euthanized at an earlier time). In spite of these limitations, we found a large increase in UCP2 protein levels in the striatum of LV-UCP2-treated rats, which were as much as 2.5-fold greater with respect to rats treated with the empty vector (Figure 3a). In the hippocampus and cerebral cortex of LV-UCP2 rats, we observed a trend to an increase in UCP2 protein levels, which, however, was not statistically significant (Figure 3d,e).

We could confirm the up-regulation of UCP2 in the striatum by performing real-time-PCR and immunohistochemical analysis. Real-time PCR was performed in the same rats used for the determination of UCP2 protein levels by immunoblotting. We found a large increase in the transcript of the gene encoding UCP2 in the striatum of LV-UCP2-treated rats, in nice agreement with immunoblot data (Figure 3b). Immunohistochemical analysis was performed in only two rats (one per group) killed at the same time, i.e., after seven weeks of the hypersodic diet. The rat treated with LV-UCP2 showed a substantial increase in UCP2 immunoreactivity in the striatum with respect to the control rat. Double fluorescent staining with anti NeuN or anti-GFAP antibodies showed UCP2 co-localized with NeuN, but not with GFAP (Figure 3c). Thus, at least in this selected animal treated with LV-UCP2, UCP2 appeared to be localized in neurons, and not in astrocytes (Figure 3c).

### 2.5. Modulation of Mitochondrial Dynamics, Antioxidant Response Elements and Inflammation in Striatum of JD-Fed SHRSP Rats Receiving LV-UCP2

**Figure 4:**
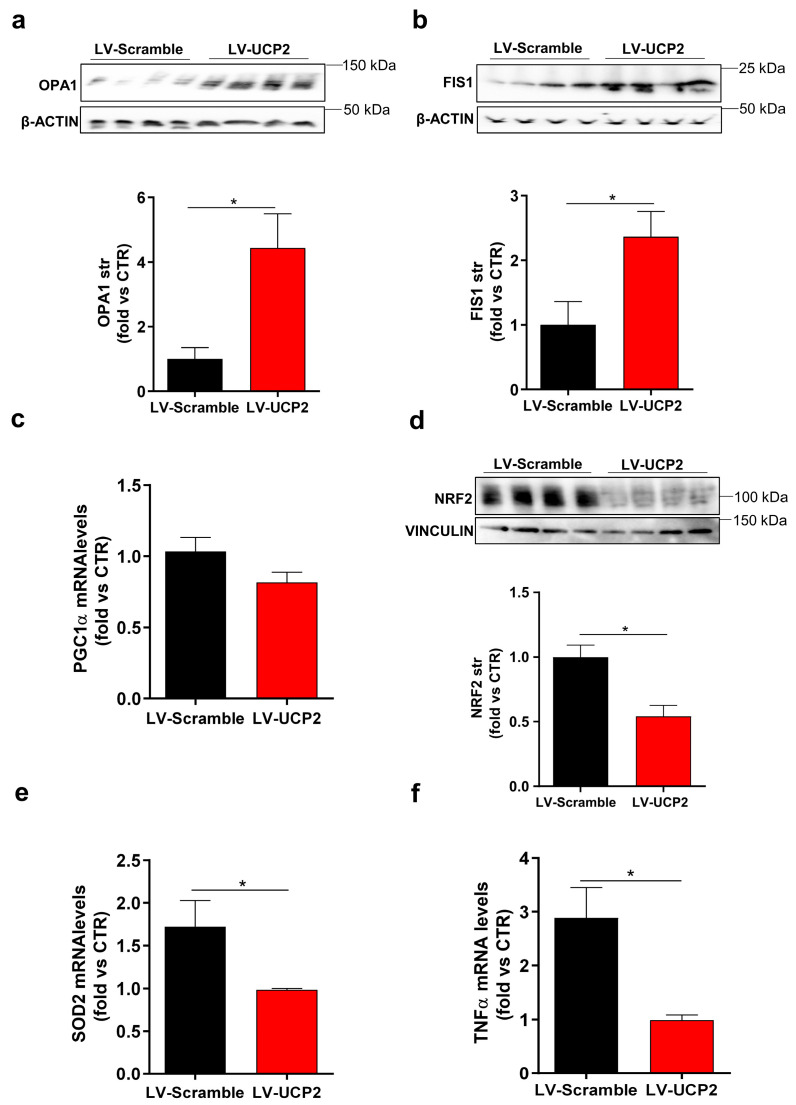
Assessment of mitochondrial dynamics, antioxidant response elements and inflammation in the striatum of JD-fed SHRSP rats receiving a single i.c.v. injection of LV-UCP2 or the empty vector (LV-Scramble). OPA1 and FIS1 protein levels are shown in (**a**,**b**), respectively. PGC1α mRNA level is shown in (**c**). Nrf2 protein level is shown in (**d**). SOD2 and TNFα mRNA levels are shown in (**e**) and (**f**), respectively. Values are means ± S.E.M. of four animals per group. * *p* < 0.05 (Student’s *t* test). Animals were killed at the time of the first stroke episode.

We investigated the impact of UCP2 overexpression on markers of mitochondrial dynamics, antioxidant response elements and inflammation in the striatum of rats receiving LV-UCP2. Compared to rats receiving an empty vector, we found a significant upregulation of optic atrophy 1 protein (OPA1) and mitochondrial fission protein 1 (FIS1), two markers of mitochondrial fusion and fission, respectively (Figure 4a,b). No significant changes were observed in the gene expression of peroxisome proliferator-activated receptor gamma coactivator 1-alpha (PGC-1α), a master regulator of mitochondrial biogenesis (Figure 4c). Then, we assessed levels of nuclear factor erythroid 2-related factor 2 (Nrf2) and superoxide dismutase 2 (SOD2), two elements orchestrating anti-oxidant responses to cell injury. We found a downregulation of both Nrf2 and SOD2 in the striatum of rats receiving LV-UCP2 (Figure 4d,e). Finally, inflammation drastically decreased following LV-UCP2 administration (Figure 4f).

## 3. Discussion

Our data (i) provide the first direct evidence that UCP2 is protective in an experimental animal model of stroke; (ii) demonstrate that the overexpression of UCP2 in the central nervous system (CNS) not only delays the occurrence of stroke, but also restrains renal damage in JD-fed SHRSP rats; and (iii) clearly dissociate UCP2 protection against hypertension-associated organ damage from changes in blood pressure. This strengthens the hypothesis that UCP2 is a key molecular player in central mechanisms of resilience to stroke and might be targeted by therapeutic intervention.

Because of our experimental design, we could assess UCP2 expression in JD-fed SHRSP rats only at the time of the first episode of stroke, which occurred at different intervals from the onset of JD. In spite of this limitation, we found a large overexpression of UCP2 in the striatum after a single i.c.v. injection of LV-UCP2, whereas only a trend to an increase in UCP2 protein levels was observed in other brain regions. This regional selectivity might reflect a preferential diffusion of the viral vector in the striatum (which is in contact with the lateral ventricle at the injection site), as well as a greater signal-to-noise, owing to the low background expression of UCP2 in the striatum [24]. However, we cannot exclude that UCP2 was also overexpressed in other brain regions at earlier times following LV-UCP2 injection.

The preferential expression of UCP2 in the striatum after i.c.v. LV-UCP2 injection made our experimental approach relevant to our stroke model, because the striatum is highly vulnerable to JD-induced pathology in SHRSP rats, as shown by extensive white matter damage and swelling in this region [25,26,27]. UCP2 was selectively localized in neurons after LV-UCP2 injection, suggesting that the direct protection of striatal neurons against mitochondrial stress and ROS formation [28] contributed to the overall effects of UCP2 overexpression that we have seen in SHRSP rats. This confers specificity to the protective activity of UCP2 in our model.

We explored the mechanism of UCP2 at the molecular level by measuring a number of factors involved in inflammation, oxidative stress, and mitochondrial homeostasis at transcriptional or protein levels. We wish to highlight that all measurements were performed in the striatum of rats killed at the first episode of stroke, and, therefore, all changes reflect long-lasting modifications that can be directly or indirectly triggered by UCP2. UCP2 overexpression was associated with an increase in both OPA1 and FIS1, two proteins related to mitochondrial fusion and fission, respectively. In contrast, a significant reduction in Nrf2 protein levels and a trend to a reduction in the transcript of PGC1α were found in UCP2 overexpressing animals. Nrf2 and PGC1α are inter alia putative molecular markers of mitochondrial biogenesis. Thus, UCP2, which is physiologically localized in the inner mitochondrial membrane, might drive changes in mitochondrial fission and fusion without increasing mitochondrial biogenesis, at least in the temporal window of our determinations. To our knowledge, changes in mitochondrial plasticity were never described before in the context of neuroprotection in the SHRSP model of stroke, and this fosters novel perspectives to be investigated for their potential therapeutic relevance.

One may argue that the reduced expression of Nrf2 protein levels and SOD2 transcript found in UCP2-overexpressing rats is not consistent with the established antioxidant activity of the two proteins. Nrf2 is a transcription factor, which, in the absence of oxidative damage, is committed to proteasomal degradation by its cytoplasmic partner Keap-1. ROS and other oxidizing agents disrupt the interaction between Nrf2 and Keap-1, reducing Nrf2 degradation. Under these conditions, Nrf2 translocates from the cytosol to the nucleus, enhancing the expression of genes encoding antioxidant proteins [29,30]. It is likely that the long-lasting reduction in Nrf2 levels observed in UCP2-overexpressing animals reflects a primary antioxidant effect of UCP2, which eliminates the need for Nrf2 activation, thus increasing intracellular Nrf2 degradation. An opposite scenario explains the overexpression of Nrf2 found in UCP2 knockout mice [31].

Similarly, a reduced production of superoxide anions, resulting from an antioxidant effect of UCP2, might explain the observed reduction of the transcript encoding SOD2, a manganese-dependent mitochondrial enzyme, which converts superoxide anions into hydrogen peroxide [32,33]. The induction of SOD2 in response to oxidative stress has been consistently demonstrated in a variety of experimental models [34,35].

Thus, the reductions in Nrf2 and SOD2 are not counterintuitive, but suggest that the antioxidant effect of UCP2 lies at the core of its mechanism of action.

UCP2 may also be protective by restraining inflammation in the striatum, as suggested by the reduction in the transcript encoding the pro-inflammatory cytokine, TNFα, in UCP2-overexpressing SHRSP rats. This additional mechanism might be highly relevant for resilience to stroke, considering the established role of inflammation in stroke-associated neuronal damage, in this and other models of cerebrovascular disorders [36].

We were surprised to find that selective UCP2 overexpression in the striatum was highly protective against early kidney damage in JD-fed SHRSP rats. A marked increase of proteinuria usually precedes stroke occurrence in the SHRSP model, as it does in humans [37,38,39]. A reduction of proteinuria is consistently observed by pharmacological or dietary interventions that prevent stroke occurrence in SHRSP [20]. Remarkably, brain overexpression of UCP2 did not change BP in SHRSP rats, raising the intriguing possibility that UCP2 expression in striatal neurons and perhaps other CNS neurons drives a protective mechanism against kidney damage, which does not involve the regulation of BP. Several lines of evidence suggest the existence of a brain-kidney cross-talk [40,41,42], which is mediated by hormones (endocrine-mediated cross-talk), baroreceptors and osmoreceptors (sensor-mediated cross-talk), and direct inter-organ innervation (neuron-mediated cross-talk) [42]. Which of these mechanisms mediate the protection against kidney damage induced by brain UCP2 expression in SHRSP rats remains to be established. Whether the delayed onset of stroke we have found in rats injected with LV-UCP2 is secondary to protection against renal damage or occurs independently of the brain-kidney axis is also unknown. Whatever the mechanism, our findings strengthen the possibility that stroke protection can be achieved in SHRSP rats without reducing BP [21,22,23]. This may hold promise for the development of neuroprotective strategies for the treatment of stroke in humans.

We observed a trend to a decrease in body weight in rats injected with LV-UCP2. This is consistent with the evidence that mitochondrial UCP2 is a key regulator of energy metabolism, and that part of its action takes place in the CNS [43]. UCP2 is a protective protein against obesity and type-2 diabetes [44,45]. It is likely that a more robust effect of brain UCP2 overexpression on body weight in our study has been masked by the strong metabolic impact of JD in SHRSP rats.

In summary, our study demonstrates, for the first time, that the brain-specific overexpression of UCP2 is protective against stroke and renal damage in JD-fed SHRSP rats, and that enhanced mitochondrial fusion and fission, and reduced oxidative damage and inflammation in the striatum, underlie the protective effect of UCP2. These data lend credit to the hypothesis that targeting UCP2 represents a valuable strategy to reduce stroke and the renal damage of hypertensive origin.

## 4. Materials and Methods

### 4.1. Experimental Design

The rats used in this study belong to the stroke-prone spontaneously hypertensive strain (SHRSP). They were kept at the animal facility of the Neuromed Institution, maintained at constant room temperature and 12 h day/night cycles, and were fed with regular rat food and water ad libitum. All animal procedures were performed by skilled and experienced experimenters to avoid unnecessary animals’ suffering caused as a result of the procedures. Fourteen male SHRSP at the age of 4 weeks underwent surgery for intracerebroventricular (i.c.v.) delivery of a lentiviral vector, containing a construct inducing a constitutive expression of UCP2 (LV-UCP2, Cellomics Technology LLC, Halethorpe, MD, USA, *n* = 7), or an empty vector used as control (LV-Scramble, Cellomics Technology LLC, *n* = 7). All studies were performed in accordance with the ARRIVE guidelines and following the Italian and European Community (Directive 2010/63/EU) for animal experiments. The protocol was approved by IRCCS Neuromed Animal Care Review Board (O.P.B.A., Organismo proposto al Benessere Animale, 139/2014-b).

### 4.2. Intracerebroventricular (i.c.v.) Injection

SHRSP rats were anesthetized with isoflurane (5% for induction and 2% for maintenance) in 100% O_2_. Isoflurane was used as an inhalation anesthetic, as it produces rapid induction and recovery from anesthesia and undergoes less biotransformation than other agents, making it a suitable choice for in vivo studies [46]. Lentiviral vectors (LV-UCP2 or LV-Scramble, 10^8^ T.U./10 µL) were injected i.c.v. in both ventricles (5 µL/5 min for each side), by using the following stereotaxic coordinates, according to the atlas of Paxinos and Watson [47]: AP: −0.4 mm; ML: ±1.2; DV: −3.5 mm.

Two weeks later, at the age of six weeks, the rats received a JD (18.7% protein, 0.37% sodium, 0.63% potassium, in addition to 1% NaCl in drinking water). Two rats (one harboring the UCP2 construct and one harboring the empty vector) were used only for the immunohistochemical analysis that was performed at the seventh week of the diet. The remaining rats (*n* = 6 for each group) were followed thereafter for occurrence of renal damage (determined by the proteinuria level in 24 h urine collections) and for stroke occurrence (diagnosed by signs of paresis, paralysis, epilepsy, sudden death), as previously reported [48,49]. Urine was collected by housing rats in individual cages at different weeks and proteinuria was determined by Bradford assay. Body weight was monitored weekly. Systolic BP level was measured by a tail-cuff sphygmomanometer, as previously reported [48]. At the time of stroke occurrence, rats were anesthetized by isoflurane inhalation for approximately 1–2 min and then sacrificed by cervical dislocation; brain and kidneys were removed for subsequent analyses.

### 4.3. Western Blot and Real-Time PCR Analysis

UCP2 protein levels were measured in the hippocampus, corpus striatum, and cerebral cortex by Western blot analysis. Rats were killed at the time of the first stroke episode, and the brain was rapidly removed and used for expression level analysis of UCP2. To do that, dissected brain regions (striatum, hippocampus and cerebral cortex) were subjected to an extraction of total RNA and proteins in order to carry out the analysis of UCP2 expression. Striatum extracts were also used for the assessment of markers of mitochondrial dynamics (OPA1, FIS1), mitochondrial biogenesis (PGC1α), antioxidant response elements (NRF2, SOD2) and inflammation (TNFα). Total RNA was extracted by trizol (Invitrogen, Carlsbad, CA). Then, 500 ng of RNA for each sample were retrotranscribed into cDNA, using the Superscript Vilo (Invitrogen) and following the manufacturer’s instructions. RTPCR was performed using the ViiA 7 Real-Time PCR System (Applied Biosystem, Foster City, CA, USA), with TaqMan methodology for UCP2 and Sybr Green Master mix (Applied Biosystem) for PGC1α, SOD2 and TNFα). The values of target mRNAs were calculated by the comparative 2^−DDCt^ method. Primers and probes (MWG Biotech, Florence, Italy) were the following: UCP2: forward 5-GAGAGTCAAGGGCTAGCGC-3, reverse 5-GCTTCGACAGTGCTCTGGTA-3, probe 5-TCAGAGCATGCAGGCATCGG-3; β-actin: forward 5-ACCCACACTGTGCCCATCTA-3, reverse 5-GCCACAGGATTCCATACCCA-3, probe 5-GCCACGCTCGGTCAGGATCTTCAT-3; PGC1α: forward 5-CGGTCTTAGCACTCAGAACC-3, reverse 5-CTGAGCAGGGACGTCTTTGT-3; SOD2: forward 5-TAACGCGCAGATCATGCAG-3, reverse 5-GTCACGCTTGATAGCCTCCA-3; TNFα: forward 5-ACAAGGCTGCCCCGACTAT-3, reverse 5-CTCCTGGTATGAAGTGGCAAATC-3; β-Actin: forward 5-GGCATCCTGACCCTGAAGTA-3, reverse 5-GGGGTGTTGAAGGTCTCAAA-3. Proteins were extracted in RIPA buffer and 50 μg for each sample were separated on SDS-PAGE and transferred to polyvinylidene difluoride membranes (Amersham, Piscataway, NJ, USA). Membranes were blocked with 5% nonfat dried milk (Biorad, Milan, Italy) for 1 hr at room temperature and then incubated overnight at +4 °C, with the following primary antibodies: UCP2 (Cell Signaling, Beverly, MA, USA, CS89326), β-actin (Santa Cruz Biotechnology, Santa Cruz, CA, 69879), OPA1 (Abcam, Cambridge, UK, ab90857), FIS1 (GeneTex, Zeeland, MI, US, GTX111010), NRF2 (abcam, ab137550), vinculin (Sigma Aldrich, Milan, IT, V9131). Signals were acquired with a Chemidoc (Biorad, Hercules, CA) and optical intensity of the bands was quantified using the Image J software.

### 4.4. Immunohistochemistry

The UCP2 overexpression in the corpus striatum was also analyzed by immunohistochemistry. For this reason, the SHRSP rats subjected to i.c.v. injection of LV-UCP2 or of LV-Scramble and then fed with a hypersodic diet for 7 weeks were anaesthetized with a mixture of ketamine:xylazine (ketamine 100 mg/kg, xylazine 5 mg/kg, i.p.) and sacrificed via a transcardiac perfusion of saline followed by paraformaldehyde (PFA, 4% in phosphate buffered saline). Dissected brains were post-fixed overnight in 4% PFA at 4 °C and then cut at the cryostat (Leica CM1520, Leica Biosystems, MI, Italy) to obtain 30 µm serial coronal sections through the striatum. Slices were incubated overnight at 4 °C with a rabbit polyclonal antibody anti-UCP2 (Proteintech, Manchester, UK; code: 11081-1-AP, 1:100) and then for 120 min at room temperature (RT) with a secondary antibody biotinylated anti-rabbit IgG (Vector Laboratories, Burlingame, CA, USA; code: BA-1000, 1:200). Finally, all slices were incubated for 120 min at RT with streptavidin Alexa-Fluor-568 conjugated (Life Technologies, Eugene, OR; code: S11226, 1:200). The blue-fluorescent 4′,6-Diamidino-2-phenylindole (DAPI) was used as nuclear couterstaining (Thermo Fisher Scientific, Waltham, MA, USA).

For double florescence analyses, the sections were incubated overnight at 4 °C with a mixture containing the following primary antibodies: rabbit polyclonal anti-UCP2 antibody (Proteintech; code: 11081-1-AP, 1:100) and a mouse monoclonal anti-glial fibrillary acid protein (GFAP, Sigma-Aldrich, SAB4501162, 1:200), or a mouse monoclonal anti-Neuronal Nuclei (NeuN, Millipore, Billerica, MA, US; code: ABN78, 1:200). Thereafter, the slices were incubated for 120 min at RT, with a mixture containing a secondary biotinylated anti-rabbit IgG (Vector, Burlingame, CA, code: BA-1000, 1:200) and an Alexa-488 conjugated anti-mouse IgG (Life Technologies, code: A21202). Finally, all slices were incubated for 120 min at RT with streptavidin Alexa-Fluor-568 conjugated (Life Technologies; code: S11226, 1:200).

### 4.5. Statistical Analysis

All continuous variables are reported as mean ± S.E.M. Log-rank and Wilcoxon statistics were used for analyzing stroke occurrence between experimental groups. Comparisons between two groups were performed using the Student’s *t* test. Statistical significance was stated at the *p* < 0.05 level. Graph Pad Prism (Ver 5.01 GraphPad Software, Inc., La Jolla, CA, USA) statistical software was used for the statistical analysis. A power analysis test was performed for sample size determination and significant data had a minimum power of 0.80 and an alpha level of 0.05. No randomization or blinding was performed in this study.

## Figures and Tables

**Table 1:** Body weight (BW) in Japanese style hypersodic diet (JD)-fed stroke-prone spontaneously hypertensive (SHRSP) rats receiving a single i.c.v. injection of either lentiviral vector encoding UCP2 (LV-UCP2) or empty vector (LV-Scramble).

	Weeks of JD							
	2	3	4	5	6	7	8	9
BW (g) LV-Scramble	163.5 ± 9.8 *n*= 6	187.8 ± 9.8 *n*= 6	196 ± 8 *n* = 6	212.2 ± 8.2 *n* = 6	210 ± 10.9 *n* = 5	NA	NA	NA
BW (g) LV-UCP2	161.5 ± 12.3 *n* = 6	176.6 ± 10.3 *n* = 6	190.6 ± 11 *n* = 6	198.83 ± 8.1 *n* = 6	200.5 ± 10.7 *n* = 6	188.16 ± 11.9 *n*= 6	193 *n* = 1	172 *n* = 1

Values are expressed as means ± SEM. NA, not available. Differences between groups were not significant.

**Table 2:** Systolic Blood Pressure (SBP) in JD-fed SHRSP rats receiving a single i.c.v. injection, of either LV-UCP2 or empty vector (LV-Scramble).

	Weeks of JD				
	4	5	6	7	8
SBP (mmHg) LV-Scramble	189 ± 1.45 *n* = 6	200 ± 3 *n* = 6	207.3 ± 9 *n* = 5	NA	NA
SBP (mmHg) LV-UCP2	186 ± 3.05 *n* = 6	201 ± 4 *n* = 6	212 ± 4 *n* = 6	198 ± 11 *n* = 6	226 *n* = 1

Values are expressed as means ± SEM. NA, not available. Differences between groups were not significant.

## Abbreviations

BP	blood pressure
BW	body weight
CNS	central nervous system
Cor	cerebral cortex
FIS1	mitochondrial fission protein 1
GFAP	glial fibrillary acid protein
Hip	hippocampus
i.c.v.	intracerebroventricular
JD	high-salt Japanese style diet
LV	lentiviral vector
NeuN	neuronal nuclei
NRF2	nuclear factor erythroid 2-related factor 2
OPA1	optic atrophy 1 protein
PGC1α	peroxisome proliferator-activated receptor gamma coactivator 1-alpha
ROS	reactive oxygen species
SHRSP	stroke-prone spontaneously hypertensive rat
SOD2	superoxide dismutase 2
Str	corpus striatum
TNFα	tumor necrosis factor alpha
UCP2	uncoupling protein-2
